# Increase in public interest concerning alternative medicine during the COVID-19 pandemic in Indonesia: a Google Trends study

**DOI:** 10.12688/f1000research.25525.2

**Published:** 2021-02-25

**Authors:** Dewi Rokhmah, Khaidar Ali, Serius Miliyani Dwi Putri, Khoiron Khoiron

**Affiliations:** 1 Department of Health Promotion and Behavior Science, Faculty of Public Health,, University of Jember, Jember, East Java, Indonesia; 2 Graduate Program of Public Health, Faculty of Medicine, Public Health and Nursing, Gadjah Mada University, Yogyakarta, Central Java, Indonesia; 3 Graduate Program of Tropical Medicine, Faculty of Medicine, Airlangga University, Surabaya, East Java, Indonesia; 4 Department of Environmental Health, Faculty of Public Health, University of Jember, East Java, Indonesia

**Keywords:** COVID-19, alternative medicine, pandemic, search activity

## Abstract

**Background:** The COVID-19 pandemic has triggered individuals to increase their healthy behaviour in order to prevent transmission, including improving their immunity potentially through the use of alternative medicines. This study aimed to examine public interest on alternative medicine during the COVID-19 pandemic using Google Trends in Indonesia.

**Methods:** Employing a quantitative study, the Spearman rank test was used to analyze the correlation between Google Relative Search Volume (RSV) of various search terms, within the categories of alternative medicine, herbal medicine and practical activity, with COVID-19 cases. In addition, time lag correlation was also investigated.

**Results:** Public interest toward alternative medicine during COVID-19 pandemic in Indonesia is dramatically escalating. All search term categories (alternative medicine, medical herbal, and alternative medicine activities) were positively associated with COVID-19 cases (p<0.05). The terms ‘ *ginger’* (r=0.6376), ‘ *curcumin’* (r=0.6550) and ‘ *planting ginger*’ (0.6713) had the strongest correlation. Furthermore, time lag correlation between COVID-19 and Google RSV was also positively significant (p<0.05).

**Conclusion:** Public interest concerning alternative medicine related terms dramatically increased after the first COVID-19 confirmed case was reported in Indonesia. Time lag correlation showed good performance using weekly data. The Indonesian Government will play an important role to provide and monitor information related to alternative medicine in order for the population to receive the maximum benefit.

## Introduction

The COVID-19 pandemic is a massive health crisis worldwide. Within seven months, it has affected 216 countries, and more than 11 million population have been infected by the SARS-COV-2 virus, which causes COVID-19 ^1^. In Indonesia, COVID-19 transmission has been reported in all provinces, with 68,226 confirmed cases recorded by July 8 ^th^ 2020 ^2^. The World Health Organization (WHO) noted that Indonesia is the third country with largest number of cases in South East Asia ^3^. Therefore, appropriate action is urgently needed to halt COVID-19 transmission among the public. 

Effenberger *et al.* ^4^ noted that the high virulence of SARS-COV-2 contributes to the super-spread of COVID-19. In addition, the large number of asymptomatic cases catalyze the intensity of the transmission among population. The pandemic has triggered a large-scale behavior change among the global population to protect their health ^5^. This may include an increase of public interest concerning alternative medicine.

Alternative medicine in Indonesia is called *Jamu* and is well-known. It is commonly composed by herbal medicines, such as ginger and curcumin, which are extracted and added to water to be drinkable. Both ingredients and other methods of *Jamu* are accessible and available to the general population of Indonesia. *Jamu* is commonly used to preserve immunity, and it has existed hereditary ^6^. Aditama ^7^ noted that 30.4% of total household in Indonesia used alternative medicine, in which this condition should be notice by Indonesian government in order to prevent alternative medicine misuse and misinformation during pandemic. Therefore, this study aimed to examine public interest concerning alternative medicines in Indonesia during the COVID-19 pandemic. Time lag scenarios were also investigated.

## Methods

This was a quantitative study using secondary data from Indonesia. The data was obtained from Google Trends using Google Relative Search Volume (RSV) and COVID-19 case data. Google RSV presents information on how many terms have been searched at a particular time using the Google search engine, i.e. the data provides information about public interest towards a particular term ^8^. A high RSV (maximum 100 points) indicates high public interest; while the lowest (0 points) shows an absence of public interest ^9^. In this study, COVID-19 cases were defined as laboratory-confirmed cases positive for SARS-COV-2 virus as reported by the Indonesian Government, in which the case number refers to total daily case of COVID-19. On June 16 ^th^ 2020, the RSV data were retrieved from January 1 ^st^ 2019 to June 6 ^th^ 2020 weekly (total of 74 weeks; 2019: weeks 1–52, 2020: weeks 53–74). The setting of Google Trend was Indonesia as country, and all categories.

### Data sources

Data for confirmed cases of COVID-19 nationwide were collected from the Indonesian Ministry of Health (MoH), where COVID-19 cases are reported daily ( https://www.covid19.go.id/peta-sebaran).

Google RSV data for Indonesia were collected from Google Trends ( https://trends.google.com) with web search as default option ^10^. Search terms were divided into three categories with subterms in each of the categories as follows: 1) alternative medicine (‘ *Jamu’* [alternative medicine]; 2) herbal medicine (‘ *tanaman obat*’ [herbal medicine], ‘ *jahe’* [ginger], ‘ *kunyit’* [curcumin]); and 3) alternative medicine activities (‘ *cara membuat jamu*’ [how to make jamu], ‘ *membuat jamu*’ [make jamu], ‘ *menanam tanaman obat*’ [planting herbal medicines], ‘ *menanam jahe*’ [planting ginger], *‘menanam kunyit’* [planting curcumin]).

The first category ‘ *Jamu’* was employed to recognize public interest toward alternative medicine during the pandemic in Indonesia; as stated before ‘ *Jamu’* is traditional alternative medicine in Indonesia used for maintaining and improving immunity. The second category (herbal medicine) was used to understand public interest on the types of medical plants being used. According to Salim and Munadi ^11^, the production of ginger and curcumin in Indonesia was the highest compared to other medicinal plants, where the consumption trend during 2011–2015 increased by 21.95% and 5.92%, respectively. Moreover, the Statistics Office of Indonesia recorded that the total harvest of ginger and curcumin on 2018 is the largest in Indonesia ^12^. Therefore, search terms of ‘ *jahe’* [ginger] and ‘ *kunyit’* [curcumin] was selected in the second category. The third category (alternative medicine activities) collected information about public interest toward performing *Jamu* and planting herbal medicines.

### Data analysis

This study followed the methodology of previous studies ^7, 13^. After checking and cleaning the data, there was no missing data noted. The data was stored in Microsoft Excel 2010, and then transferred to STATA v13 (College Station, TX, USA) for analysis. Google RSV data was available weekly, and therefore COVID-19 case data was also analyzed weekly.

The data was not normally distributed, so Spearman rank test was used to examine the correlation between Google RSV and COVID-19 cases. Time lag correlation between Google RSV and COVID-19 was also analyzed, where the procedure referred to Husnayain *et al.* ^13^ and Torres-Reyne ^14^. The significance level was set at 0.05.

## Results

### COVID-19 cases and Google RSV

**Figure 1.**
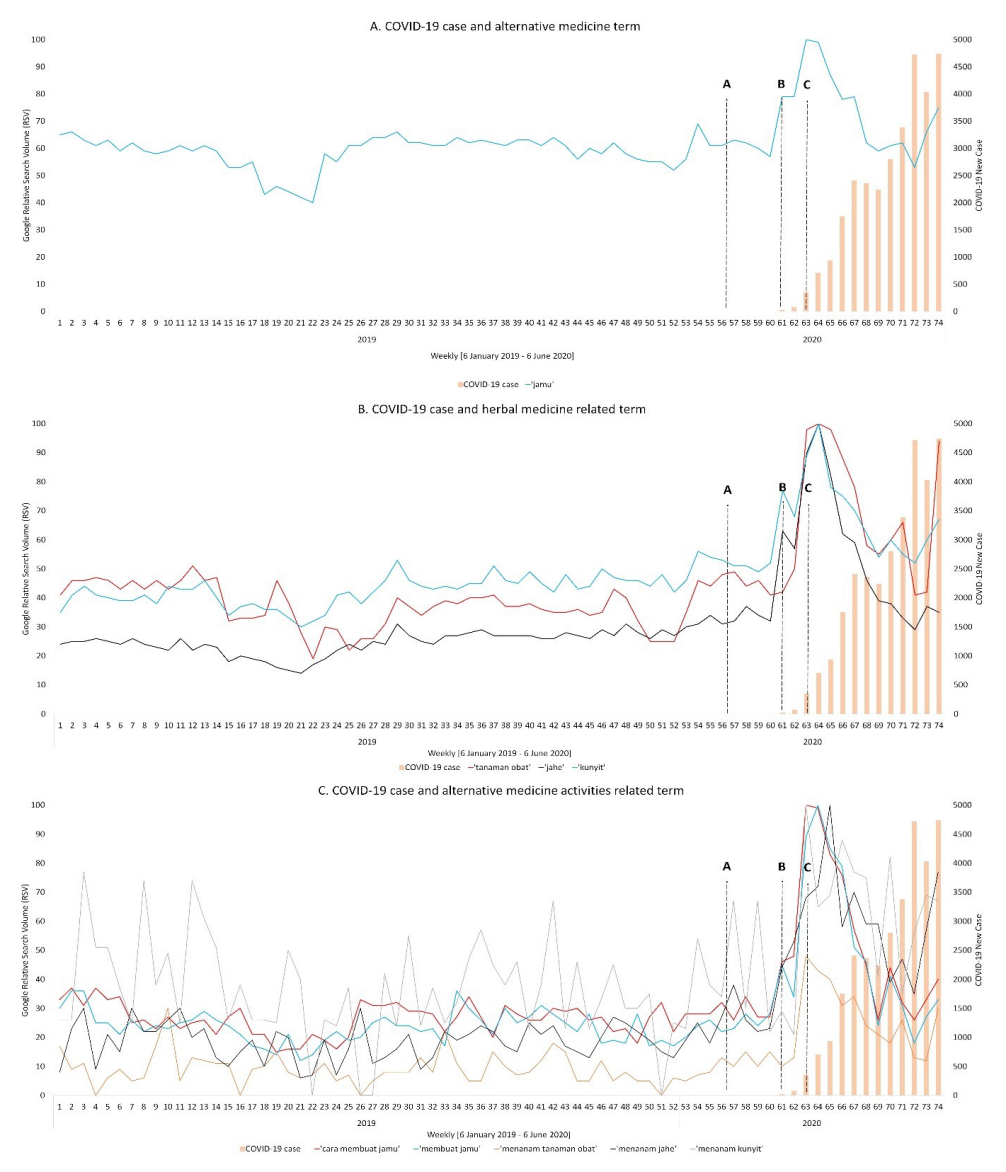
Google Relative Search Volume and COVID-19 new cases in Indonesia. COVID-19 cases compared with ( **A**) *‘*Jamu’ [alternative medicine] search term; ( **B**) herbal medicine search terms (‘ *tanaman obat*’ [herbal medicine], ‘ *jahe’* [ginger], ‘ *kunyit’* [curcumin]); ( **C**) alternative medicine activities search terms (‘ *cara membuat jamu*’ [how to make jamu], ‘ *membuat jamu*’ [make jamu], ‘ *menanam tanaman obat*’ [planting herbal medicines], ‘ *menanam jahe*’ [planting ginger], and ‘ *menanam kunyit’* [planting curcumin]). Letters: A, January 30 ^th^ 2020: COVID-19 declared as Public Health Emergency of International Concern; B, March 2 ^nd^ 2020: first imported case was reported in Indonesia; C, March 16 ^th^ 2020: social distancing declared by Indonesian Government.

The pattern of COVID-19 case and Google RSV in Indonesia is visualized in Figure 1. Since the first confirmed COVID-19 case was reported in Indonesia on March 2 ^nd ^2020 (week 61 of this study), COVID-19 cases have been increasing in Indonesia. According to the MoH, 30,514 confirmed cases of COVID-19 were reported during 14 weeks (March 2 ^nd^–June 6 ^th^ 2020); mean weekly cases were recorded as ~315 cases. 

RSV of ‘ *Jamu’* [alternative medicine] from week 1 until week 60 was 40–60 points, with search activity increasing from week 61 (March 1 ^st^-7 ^th ^2020). The highest RSV score for this search term was in week 63 with 100 points ( Figure 1A). The RSV of ‘ *tanaman obat*’ [herbal medicine], ‘ *jahe’* [ginger], and ‘ *kunyit’* [curcumin] before the pandemic (week 1–60) was 19–49 points, with the RSV dramatically increasing from week 61 (42–79 points). The peak for all herbal medicine search terms was found in week 64 (100 points) ( Figure 1B).

A similar trend is shown for alternative medicine activities search terms ( Figure 1C). Before the pandemic (week 1–60) these terms had an RSV of 0–36 points. In week 61, the RSV increases ~2 fold higher. The term ‘ *cara membuat jamu*’ [how to create jamu] and ‘ *membuat jamu*’ [create jamu] reached their peak on week 63 (100 points) and 64 (100 points), respectively. Meanwhile, the peak for ‘ *menanam jahe*’ [planting ginger] and ‘ *menanam kunyit’* [planting curcumin] was recorded on week 65 and week 63, respectively, with 100 points. The peak for ‘ *menanam tanaman obat*’ [planting herbal medicines] reached its peak on week 63 (similar to ‘ *cara membuat jamu*’ [how to create jamu]) with the highest score of 48 points.

### Correlation analysis results

Table 1 displays the correlation between COVID-19 cases and Google RSV in Indonesia. All search term categories (alternative medicine, herbal medicine, and alternative medicine activities) are positively correlated with COVID-19 cases (p<0.05). The terms ‘ *jahe’* [ginger] (r=0.6376), ‘ *kunyit’* [curcumin] (r=0.6550) and ‘ *menanam jahe*’ [planting ginger] (r=0.6713) have the strongest correlation towards COVID-19 new cases in Indonesia. Based on a time lag scenario, the correlation between COVID-19 cases and Google RSV showed good performance with weekly data, where all search terms are significant (p<0.05). In the time lag scenario, a strong correlation is also found for the terms ‘ *jahe’* [ginger], ‘ *kunyit’* [curcumin], and ‘ *menanam jahe*’ [planting ginger] (r>0.6; p<0.05).

**Table 1.** Correlation between Google Relative Search Volume and COVID-19 cases in Indonesia.

Search term	Weeks						
	lag -3	lag -2	lag -1	lag 0	lag 1	lag 2	lag 3
**Alternative medicine**							
‘ *Jamu’* [alternative medicine]	0.4351 ***	0.3858 **	0.3917 **	0.4028 ***	0.3165 **	0.3113 **	0.3032*
**Herbal medicine**							
‘ *tanaman obat*’ [herbal medicine]	0.5231 ****	0.5474 ****	0.5648 ****	0.5643 ****	0.5839 ****	0.5408 ****	0.5330 ****
‘ *jahe’* [ginger]	0.6362 ****	0.6306 ****	0.6289 ****	0.6376 ****	0.5806 ****	0.5668 ****	0.5422 ****
‘ *kunyit’* [curcumin]	0.6096 ****	0.6115 ****	0.6238 ****	0.6550 ****	0.5974 ****	0.5839 ****	0.5623 ****
**Alternative medicine activities**							
‘ *cara membuat jamu*’ [how to make jamu]	0.5324 ****	0.4589 ****	0.5101 ****	0.5127 ****	0.4573 ****	0.4410 ****	0.4360 ***
‘ *membuat jamu*’ [make jamu]	0.5531 ****	0.5082 ****	0.5592 ****	0.4874 ****	0.4525 ***	0.4236 ***	0.4132 ***
‘ *menanam tanaman obat*’ [planting herbal medicine]	0.5212 ****	0.5312 ****	0.5609 ****	0.5690 ****	0.5778 ****	0.5583 ****	0.5394 ****
‘ *menanam jahe*’ [planting ginger]	0.5699 ****	0.5802 ****	0.6117 ****	0.6713 ****	0.6253 ****	0.6174 ****	0.6052 ****
‘ *menanam kunyit*’ [planting curcumin]	0.2830 *	0.3019 *	0.3146 ***	0.4187 ***	0.4076 ***	0.5019 ****	0.4790 ****

*significant ( *p*<0.05); **significant ( *p*<0.01); ***significant (p<0.001); ****significant (p<0.0001)

## Discussion

Since the first COVID-19 confirmed case was reported on March 2 ^nd^ 2020 (week 61), there have been a dramatic increases in cases in Indonesia. The mean weekly cases of COVID-19 is ~315 case ( Figure 1), and we noted the highest case load reported on week 74 (4741 cases). We also show in our data that COVID-19 cases in Indonesia have increased by ~305% within 14 weeks (30,514 cases; Figure 1). This indicates a super-spread of COVID-19 in Indonesia. The high population and population mobility may take an essential role in intense COVID-19 transmission ^15, 16^. 

Alternative medicine is one option for individuals to maintain and increase their immunity during the COVID-19 pandemic. In our study, we found that the search activity of alternative medicine-related terms, including herbal medicine and activities surrounding alternative medicine, was low and steady before the pandemic (weeks 1–60). This was even though a Public Health Emergency of International Concern had been declared by the WHO on January 30 ^th^ 2020 (week 56). Interestingly, only after the first COVID-19 confirmed case in Indonesia was announced on week 61 did the search activity dramatically increased. Most of the search terms looked at in this study reached their peak on week 63–64, after which social distancing issue has been established in Indonesia (on March 16 ^th^ 2020) ^17^. The alternative medicine issue also appeared among the public around March 13 ^th^ – 16 ^th^ (week 63) during the pandemic. In this period, the President of Indonesia claimed that herbs can fight COVID-19, which may have increased public interest toward alternative medicine ^18^.

In this study, all search terms were associated positively with COVID-19 cases in Indonesia (p<0.05), and the correlation coefficient showed moderate. This indicated that increasing COVID-19 cases elevated the public interest concerning alternative medicine. A similar result was also shown with the time lag scenario, where all search terms were positively associated with COVID-19 cases (p<0.05). This finding shows that there was an increase in search activities 1–3 weeks after and before the increase of COVID-19 cases in Indonesia. However, a strong correlation is detected at the present time (lag 0) compare to time lag scenario, particularly for the herbal medicine category. This study found that correlation analysis using weekly data of Google RSV compared to COVID-19 new cases in Indonesia showed good performance, which is collaborated by previous studies ^9, 19– 23^. In addition, the moderate correlation occurs due to several factors, particularly public interest on alternative medicine term is high by intense exposure from mass media.

The trend of Google RSV for all search terms was higher during the pandemic. This indicates increasing public interest toward alternative medicine during the pandemic in Indonesia. This finding collaborates to Mavragani and Ochoa ^24^, where monitoring online queries can provide insight into human behavior. Wise *et al.* ^25^ noted that awareness of the public related to the COVID-19 pandemic is elevated due to the risk posed by the virus, and the large number of available information sources serves to reinforce their protective behavior. Galankis ^26^ also reported that the public tend to search for information related to health either short- or long-term during the pandemic. Besides, Yuan *et al.* ^27^ reported association of internet search-interest with COVID-19 daily incidence and death in USA.

As a telemedicine, smartphone technology has important role in the current COVID-19 pandemic ^28^. It contains web search that is a valuable resource for individuals and communities seeking health information or disease outbreaks, in which the search question includes geographical and timely information ^29^. Google, as one of the search engines, will construct digital traces. Google Trends data are highly related to traditional surveillance data ^30, 31^. It provides valuable source of information to investigate changes in disease patterns and health dynamics within populations using digital traces ^32^. Indonesia itself has 53.7% of global internet usage ^33^, and Google utilized is reported to be considerable at 98.3% ^34^. Therefore, Google Trend became great alternative surveillance in Indonesia.

The Indonesian Government plays an important role in the high public interest toward alternative medicine during the pandemic. Actions concerning monitoring and providing valid information regarding alternative medicine to the public are urgently needed. These actions should prevent misuse of medical herbal among the public. In addition, information could be used to empower communities to provide self-remedial source at a household level, such as planting herbal medicines.

There are limitations in this study, namely: 1) The data time range is weekly. This condition occurs due to default setting in Google Trend, where the author retrieved the RSV data from January 1 ^st^ 2019 to June 6 ^th^ 2020, and the RSV appears weekly. 2) The author analyzes the trend of public interest on alternative medicine term in the early pandemic (14 weeks), where this is the latest COVID-19 update case since this study was written. Therefore, the author recommend further study is needed to analyze the trend of public interest on alternative medicine term during pandemic by using daily data on the current situation in Indonesia, with time series analysis. In addition, study to examine the government action to prevent misinformation and misused on alternative medicine-used during pandemic is also needed.

An interesting study also found that the Google Trend study cannot provide sociodemographic feature of user who search in Google, in which this condition may become challenging to examine public interest on particular search term by stratification of the population condition ^35, 36^.

## Conclusion

Public interest on alternative medicine related-terms has dramatically increased during the early COVID-19 pandemic in Indonesia. Search terms relating to alternative medicine, herbal medicines and activities surrounding alternative medicines correlate positively with an increase of COVID-19 cases in Indonesia. This study recommends that the Indonesian Government take an active role in informing the public about alternative medicines, and monitoring and providing valid information. This may empower households to produce medical herbs independently.

## Data availability

### Underlying data

COVID-19 case data available from: https://www.covid19.go.id/peta-sebaran

Google Trend data available from: https://trends.google.com/. Search terms and other parameters are provided in the text.

Mendeley: Public interest on alternative medicine during pandemic in Indonesia, http://dx.doi.org/10.17632/fv7tprb24j.1 ^37^.

Data are available under the terms of the Creative Commons Attribution 4.0 International license (CC-BY 4.0).
