# Supplementary material for: Increase in public interest concerning alternative medicine during the COVID-19 pandemic in Indonesia: a Google Trends study
Source: F1000Res. 2021 Feb 25;9:1201. Originally published 2020 Oct 6. [Version 2] doi: 10.12688/f1000research.25525.2 (PMC7879216; doi:10.12688/f1000research.25525.2)
Supplement: Peer review history

# Peer review: Reviewer response for version 2

**DOI:** 10.5256/f1000research.54695.r80246

**Published:** 2021-02-26

**Authors:** Sinan Kardeş

**Affiliations:** 1 Department of Medical Ecology and Hydroclimatology, Istanbul University, Istanbul, Turkey

Thank you for clarifications.

Is the work clearly and accurately presented and does it cite the current literature?

Yes

If applicable, is the statistical analysis and its interpretation appropriate?

Yes

Are all the source data underlying the results available to ensure full reproducibility?

Yes

Is the study design appropriate and is the work technically sound?

Yes

Are the conclusions drawn adequately supported by the results?

Yes

Are sufficient details of methods and analysis provided to allow replication by others?

Yes

Reviewer Expertise:

Balneotherapy; Rheumatic and Musculoskeletal Diseases; Google Trends

I confirm that I have read this submission and believe that I have an appropriate level of expertise to confirm that it is of an acceptable scientific standard.

# Peer review: Reviewer response for version 2

**DOI:** 10.5256/f1000research.54695.r80245

**Published:** 2021-02-25

**Authors:** Seyed Mohammad Ayyoubzadeh

**Affiliations:** 1 Department of Health Information Management, School of Allied Medical Sciences, Tehran University of Medical Sciences, Tehran, Iran

The authors have addressed the points raised in my previous review.

Is the work clearly and accurately presented and does it cite the current literature?

Partly

If applicable, is the statistical analysis and its interpretation appropriate?

Yes

Are all the source data underlying the results available to ensure full reproducibility?

Yes

Is the study design appropriate and is the work technically sound?

Yes

Are the conclusions drawn adequately supported by the results?

Yes

Are sufficient details of methods and analysis provided to allow replication by others?

Yes

Reviewer Expertise:

Medical Informatics, eHealth, mHealth, IoT, smartphone apps, data mining, CDSS

I confirm that I have read this submission and believe that I have an appropriate level of expertise to confirm that it is of an acceptable scientific standard.

# Peer review: Reviewer response for version 1

**DOI:** 10.5256/f1000research.28170.r78465

**Published:** 2021-02-11

**Authors:** Sinan Kardeş

**Affiliations:** 1 Department of Medical Ecology and Hydroclimatology, Istanbul University, Istanbul, Turkey

It is an important study. Congratulations. I have few points:

1. Please add a paragraph mentioning Google Trends is used in COVID-19 studies to highlight its usefulness in health studies. You may refer following papers ^1^, ^2^, ^3^.
2. In Methods: Please add when the Google Trends database searched: E.g. On 11 February 2021, Google Trends data were retrieved from...
3. In Methods: Google Trends search: The time period was mentioned; but please mention the other filters: country (Indonesia?), categories (All categories or Health?).
4. r > 0.7 indicates strong correlation. Please mention this and revise thoroughly the manuscript particularly for the word 'strong correlation'. 
5. In the Results: Please change the name of subheading "Statistical analysis". It was generally used in the Methods section. You may change "Correlation analysis results", or something appropriate.
6. For your further studies, I suggest to consider performing time series analysis.
7. Please discuss limitations of the study. The authors may refer following papers for limitations ^4^, ^5^. 
8. Conclusion: Please add "early": "during the early COVID-19 pandemic".

Is the work clearly and accurately presented and does it cite the current literature?

Yes

If applicable, is the statistical analysis and its interpretation appropriate?

Yes

Are all the source data underlying the results available to ensure full reproducibility?

Yes

Is the study design appropriate and is the work technically sound?

Yes

Are the conclusions drawn adequately supported by the results?

Yes

Are sufficient details of methods and analysis provided to allow replication by others?

Yes

Reviewer Expertise:

Balneotherapy; Rheumatic and Musculoskeletal Diseases; Google Trends

I confirm that I have read this submission and believe that I have an appropriate level of expertise to confirm that it is of an acceptable scientific standard.

## References

[1] : Public interest in spa therapy during the COVID-19 pandemic: analysis of Google Trends data among Turkey. *Int J Biometeorol*.2021; 10.1007/s00484-021-02077-1 10.1007/s00484-021-02077-1 33442780PMC7805426

[2] : Population-level interest in anti-rheumatic drugs in the COVID-19 era: insights from Google Trends. *Clin Rheumatol*.2020; 10.1007/s10067-020-05490-w 10.1007/s10067-020-05490-w 33130946PMC7603411

[3] : Public interest in rheumatic diseases and rheumatologist in the United States during the COVID-19 pandemic: evidence from Google Trends. *Rheumatol Int*.41(2) : 10.1007/s00296-020-04728-9 329-334 10.1007/s00296-020-04728-9 33070255PMC7568841

[4] : Seasonal variation in the internet searches for psoriasis. *Arch Dermatol Res*.2019;311(6) : 10.1007/s00403-019-01921-0 461-467 10.1007/s00403-019-01921-0 31025101

[5] : Seasonality of bruxism: evidence from Google Trends. *Sleep Breath*.2019;23(2) : 10.1007/s11325-019-01787-6 695-701 10.1007/s11325-019-01787-6 30790220

# Response

**Published:** 2021-02-12

**Authors:** Dewi Rokhmah

**Affiliations:** University of Jember, Indonesia

Dear Dr. **Sinan Kardeş,**

 Thanks for your constructive review. This is our feedback for your review.

 1. Please add a paragraph mentioning Google Trends is used in COVID-19 studies to highlight its usefulness in health studies. You may refer following papers ^1^, ^2^, ^3^.

**Response:**

 Sure, we have added the following literature as our references (discussion section). Thank you for your suggestion

 2. In Methods: Please add when the Google Trends database searched: E.g. On 11 February 2021, Google Trends data were retrieved from...

**Response:**

 Sure, we applied this recommendation.

 3. In Methods: Google Trends search: The time period was mentioned; but please mention the other filters: country (Indonesia?), categories (All categories or Health?).

**Response:**

 Sure, we applied this recommendation

 4. r > 0.7 indicates strong correlation. Please mention this and revise thoroughly the manuscript particularly for the word 'strong correlation'. 

**Response:**

 Sure.

 5. In the Results: Please change the name of subheading "Statistical analysis". It was generally used in the Methods section. You may change "Correlation analysis results", or something appropriate.

**Response:**

 Sure.

 6. For your further studies, I suggest to consider performing time series analysis.

**Response:**

 Sure, it become our recommendation for further study. Thank you for your suggestion.

 7. Please discuss limitations of the study. The authors may refer following papers for limitations ^4^, ^5^. 

**Response:**

 Thank you. We had added this literature as our reference in order to describe limitation on our study.  

 8. Conclusion: Please add "early": "during the early COVID-19 pandemic".

**Response:**

 Sure.

 In the latest version of our manuscript is revised based on your recommendation. Thank you for your review.

# Peer review: Reviewer response for version 1

**DOI:** 10.5256/f1000research.28170.r78462

**Published:** 2021-02-05

**Authors:** Seyed Mohammad Ayyoubzadeh

**Affiliations:** 1 Department of Health Information Management, School of Allied Medical Sciences, Tehran University of Medical Sciences, Tehran, Iran

The paper analyzed if the public interest in alternative medicine had changed during the COVID-19 pandemic in Indonesia using Google Search data provided by Google Trends. The authors used the Spearman rank test for performing the statistical test. The result showed that the public interest in alternative medicine has increased during COVID-19.

 The study has cited related literature analyzing Google Trends in COVID-19. However, It will be great if the authors could add some references about alternative medicine in the introduction section.

 The study design seems appropriate, choosing a reasonable time range for the analysis and performing a suitable statistical test for the analysis.

 Please remove the sentence *"Currently, no vaccine has been developed for COVID-19".*

 The URL for google trends is " https://trends.google.com/" not *"www.trends.google.com"* mentioned in the data source.

 I couldn't find the data from the mentioned URL: https://www.kemkes.go.id/article/view/20031900002/Dashboard-Data-KasusCOVID-19-di-Indonesia.html

Is the work clearly and accurately presented and does it cite the current literature?

Partly

If applicable, is the statistical analysis and its interpretation appropriate?

Yes

Are all the source data underlying the results available to ensure full reproducibility?

Yes

Is the study design appropriate and is the work technically sound?

Yes

Are the conclusions drawn adequately supported by the results?

Yes

Are sufficient details of methods and analysis provided to allow replication by others?

Yes

Reviewer Expertise:

Medical Informatics, eHealth, mHealth, IoT, smartphone apps, data mining, CDSS

I confirm that I have read this submission and believe that I have an appropriate level of expertise to confirm that it is of an acceptable scientific standard, however I have significant reservations, as outlined above.

# Response

**Published:** 2021-02-12

**Authors:** Dewi Rokhmah

**Affiliations:** University of Jember, Indonesia

Dear Dr. **Seyed Mohammad Ayyoubzadeh**,

 Thank you for your constructive review. This is our feedback for your review:

 1. We had added some references about alternative medicine in the introduction section.

 2. The sentence “ *Currently, no vaccine has been developed for COVID-19” *has been remove in the article.

 3. We updated the data source of google trend in the article to " https://trends.google.com/"

 4. The following website ( https://www.kemkes.go.id/article/view/20031900002/Dashboard-Data-KasusCOVID-19-di-Indonesia.html) is the oldest version of surveillance COVID-19 website from Indonesian Ministry of Health (MoH). The data of COVID-19 case in Indonesia is also found in https://covid19.go.id/peta-sebaran, which is the data is integrated with the Indonesian Ministry of Health (MoH). The website ( https://covid19.go.id/peta-sebaran) itself is published by COVID-19 Response Acceleration Task Force of Indonesia (RATF) that is directly created by Indonesian President to combat COVID-19 in Indonesia. Therefore, in order to create accessible data source in this article, we consider to use the data from RATF which is the data is integrate with previous website from MoH.

# Peer review: Reviewer response for version 1

**DOI:** 10.5256/f1000research.28170.r73844

**Published:** 2020-11-11

**Authors:** Lanjing Zhang

**Affiliations:** 1 Department of Biological Sciences, Rutgers University, Newark, NJ, USA

This is an interesting article focused on the links between google search trend and daily incidence of COVID-19 in Indonesia. The findings appear novel since my search of the literature shows no similar works in the Pubmed. However, I have the following concerns:

 Major: 

1. The correlation coefficients appeared moderate (about 0.5-0.6). This low degree of correlation should be addressed. One of the approaches is to correlate the keyword with the google trend. If such a correlation is moderate (in Indonesia), the correlation coefficient become acceptable. Of course, some discussions are needed even so. 
2. Literature review is less comprehensive. It could be improved by citing related articles ^1^, ^2^, ^3^ and others. 
3. The authors may compare the trends of search interest in these alternative medicine terms, whose change may have a better correlation with the COVID-19 incidence trends.
4. Do the symptoms correct with COVID-19 daily incidence? They may correlate better than these alternative medicine terms. 
5. Not sure why Spearman ranked test was used. In my view, Pearson's rho may be a better choice. 

Minor:

1. It will be more helpful if the authors could discuss more on how direct use of these trends could improve disease surveillance and prevention.
2. Detailed p values should be provided even they are smaller than .05. 
3. The case number is probably meant daily new cases or daily incidence. Please use the more precise terms. 
4. The total case number should be updated since the one in the text is published 4 months. The number may have been doubled. 

Is the work clearly and accurately presented and does it cite the current literature?

Partly

If applicable, is the statistical analysis and its interpretation appropriate?

Partly

Are all the source data underlying the results available to ensure full reproducibility?

Yes

Is the study design appropriate and is the work technically sound?

Yes

Are the conclusions drawn adequately supported by the results?

Yes

Are sufficient details of methods and analysis provided to allow replication by others?

Partly

Reviewer Expertise:

clinical epidemiology, statistical methodology

I confirm that I have read this submission and believe that I have an appropriate level of expertise to confirm that it is of an acceptable scientific standard, however I have significant reservations, as outlined above.

## References

[1] : Trends and Prediction in Daily New Cases and Deaths of COVID-19 in the United States: An Internet Search-Interest Based Model. *Explor Res Hypothesis Med*.2020;5(2) : 10.14218/ERHM.2020.00023 1-6 10.14218/ERHM.2020.00023 32348380PMC7176069

[2] : Validation: The Use of Google Trends as an Alternative Data Source for COVID-19 Surveillance in Indonesia. *Asia Pac J Public Health*.32(6-7) : 10.1177/1010539520940896 368-369 10.1177/1010539520940896 32643957PMC7521008

[3] : COVID-19 and applications of smartphone technology in the current pandemic. *Diabetes Metab Syndr*.14(5) : 10.1016/j.dsx.2020.05.033 733-737 10.1016/j.dsx.2020.05.033 32497963PMC7248636

# Response

**Published:** 2021-02-12

**Authors:** Dewi Rokhmah

**Affiliations:** University of Jember, Indonesia

Dear Dr. **Lanjing Zhang**,

 Thank you for your constructive review. This is our response:

 Major: 

 1. The correlation coefficients appeared moderate (about 0.5-0.6). This low degree of correlation should be addressed. One of the approaches is to correlate the keyword with the google trend. If such a correlation is moderate (in Indonesia), the correlation coefficient become acceptable. Of course, some discussions are needed even so. 

**Response:**

 Sure. In the latest version, we informed this condition in discussion section.

 2. Literature review is less comprehensive. It could be improved by citing related articles ^1^, ^2^, ^3^ and others. 

**Response:**

 Sure. Thank you for your suggestion.

 3. The authors may compare the trends of search interest in these alternative medicine terms, whose change may have a better correlation with the COVID-19 incidence trends.

**Response:**

 Sure, it is.

 In this article, we classified the terms of alternative medicine into three categories in order to obtain proper information about public interest on alternative medicine during pandemic, namely: 1) alternative medicine, 2) herbal medicine, and 3) alternative medicine activities.

 Group 1 (alternative medicine) represent the term of alternative medicine itself in Bahasa or synonym (“jamu” [alternative medicine]).

 Group 2 (herbal medicine) represent the term of the type of herbal medicine that commonly used in public ( *tanaman obat*’ [herbal medicine], ‘ *jahe’* [ginger], ‘ *kunyit’* [curcumin]).

 Group 3 (alternative medicine activities) represent the term of the public activities to provide or create alternative medicine ((‘ *cara membuat jamu*’ [how to make jamu], ‘ *membuat jamu*’ [make jamu], ‘ *menanam tanaman obat*’ [planting herbal medicines], ‘ *menanam jahe*’ [planting ginger], *‘menanam kunyit’ *[planting curcumin]).  

 The author performed not only statistical test, but also graphical analysis in this study. The statistical test was used to assess the correlation between alternative medicine terms to COVID-19 daily case. On the other hand, graphical analysis was also performed to analyse the trend of public search/ public interest on alternative medicine terms to COVID-19 daily case. In addition, graphical analysis could also compare the trend of search interest in the alternative medicine term. 

 4. Do the symptoms correct with COVID-19 daily incidence? They may correlate better than these alternative medicine terms.

**Response:**

 As we mentioned in the article, the objective of the study is to analyze the public interest on alternative medicine during COVID-19 pandemic, in which alternative medicine is well-known in Indonesia. The hypothesis of this study is the public interest should be rising during pandemic condition, where this condition occur related to health seeking behaviour among community during pandemic. In addition, Google Trend study is used to obtain information of public interest.

 This study is necessary, where the study supply information about public interest on alternative medicine to Indonesian Government during pandemic, where Indonesian Government may play important role to provide and monitor appropriate information related to the use of alternative medicine during COVID-19 pandemic.   

 5. Not sure why Spearman ranked test was used. In my view, Pearson's rho may be a better choice. 

**Response:**

 In this article, we used spearman ranked test due to the result of normality test of the data, where the data is not normally distributed. Therefore, spearman rank test is fit to assess the correlation between dependent variable to independent variable ^1^. In addition, if we referred to Mavragani et al (2018) ^2^ in her systematic review of Google Trend study, the spearman rank test is also the second most used to examine the correlation in Google Trends study.

^1 ^Sarmento, D. no year. Chapter 22: Correlation Types and When to Use Them. Available: https://ademos.people.uic.edu/Chapter22.html#22_spearman_correlation (accessed on 10-02-2021)

^2^ Mavragani, A., Ochoa, G., Tsagarakis, K.P. 2018. Assessing the methods, tools, and statistical approaches in google trends research: systematic review. Journal of Medical Internet. 20(11):e270

 Minor:

 1. It will be more helpful if the authors could discuss more on how direct use of these trends could improve disease surveillance and prevention.

**Response:**

 Sure, this recommendation has been applied in this study, where we added related information in discussion section

 2. Detailed p values should be provided even they are smaller than .05. 

**Response:**

 Sure.

 According to statistical test, the range of p-value was between 0.0194 – 0.0000. But, mostly the p-value score is 0.0000. Therefore, we create 4 groups/classifications in order to present the specific p-value:

-
  *           : p<0.05
  **          : p<0.01
  ***        : p<0.001
  ****       : p <0.0001

3. The case number is probably meant daily new cases or daily incidence. Please use the more precise terms. 

**Response:**

 The case number refers to total daily case of COVID-19 in Indonesia. In the latest version of our article, we insert this information in methodology.  

 4. The total case number should be updated since the one in the text is published 4 months. The number may have been doubled. 

**Response:**

 This issue become limitation of our study, and we had declared this limitation on discussion section in which the author also mentioned recommendation for further study.
